# Research Progress on Optimization Strategies for Low-Temperature Performance of Sodium-Ion Batteries

**DOI:** 10.3390/ma19173634

**Published:** 2026-08-26

**Authors:** Pan Li, Xudong Wang, Wanli Xu, Youjie Zhou, Long Huang, Jinmao Chen

**Affiliations:** Military New-Energy Technology Research Department, Systems Engineering Institute, AMS, PLA, Beijing 102300, China; lipan1202@163.com (P.L.); xuwl06@163.com (W.X.); zhoujie_76@126.com (Y.Z.); hlong0000@163.com (L.H.)

**Keywords:** sodium-ion batteries, low-temperature performance, electrolyte engineering, electrode materials, interface regulation

## Abstract

**Highlights:**

- Smaller Stokes radius and lower desolvation energy of Na^+^ versus Li^+^ provide unique thermodynamic benefits for low-temperature operation.
- Electrolyte engineering, cathode/anode material design, and interface optimization are systematically reviewed as core performance boosters.
- Ultra-low-temperature electrolytes, multi-scale architectures, pre-sodiation, and machine learning are highlighted as next-frontier research directions.

**Abstract:**

Sodium-ion batteries (SIBs) have emerged as a highly promising candidate for large-scale energy storage and low-temperature (LT) applications, featuring abundant raw materials, low cost and working mechanisms analogous to lithium-ion batteries (LIBs). Although the ionic radius of Na^+^ is slightly larger than that of Li^+^, their smaller Stokes radius and lower desolvation energy barrier endow SIBs with unique thermodynamic advantages in LT environments. However, under extremely LT conditions, issues such as a sharp increase in electrolyte viscosity, sluggish desolvation kinetics, lattice distortion and detrimental phase transitions in electrode materials, as well as instability at the electrode–electrolyte interface, collectively constrain the LT electrochemical performance of SIBs. Most existing literature merely conduct fragmented and decoupled summaries focusing on a single component (electrolyte, cathode or anode), lacking systematic elucidation of the multi-factor coupled degradation mechanism under LT conditions and holistic evaluation of multi-dimensional modification strategies. To fill this research gap, this work systematically elaborates the intrinsic LT degradation mechanism of SIBs driven by multi-physical-field coupling. From four core perspectives, including electrolyte engineering, cathode modification, anode structural construction and precise interface regulation, we comprehensively summarize mainstream technical systems for LT performance enhancement at the current stage, and thoroughly analyze the working principle, technical merits and inherent limitations of various modification approaches. Finally, the future development directions of SIBs are prospected on the basis of previous research, aiming to provide systematic and scientific theoretical guidance for in-depth mechanism exploration and industrial technological upgrading of wide-temperature-range, high-performance SIBs.

## 1. Introduction

With the profound advancement of global energy structure transformation, large-scale energy storage technologies are receiving unprecedented strategic attention. LIBs, owing to their high energy density and mature industrial chain, have dominated the consumer electronics and electric vehicle markets. However, the crustal abundance of lithium resources is only about 0.0065%, and their distribution is extremely uneven, posing long-term challenges to the cost stability and supply chain security of LIBs. Against this backdrop, SIBs have rapidly emerged as a popular candidate for next-generation electrochemical energy storage technologies due to the abundance of sodium resources (crustal abundance ~2.65%), low cost, and the “rocking-chair” ion storage mechanism similar to that of LIBs [1,2].

Table 1 shows the main comparison between LIBs and SIBs. Of particular interest is the potential advantage of SIBs over LIBs in LT environments. Although the ionic radius of Na^+^ (0.102 nm) is slightly larger than that of Li^+^ (0.076 nm), its solvated ions in electrolytes exhibit a smaller Stokes radius and a significantly lower desolvation energy barrier, intrinsically endowing SIBs with faster ion transport capabilities and superior rate performance at LTs [3]. Additionally, the lower reactivity of sodium and the reduced risk of thermal runaway further enhance their safety and adaptability in cold-climate applications [4]. However, a significant gap remains between theory and practice—the current LT electrochemical performance of SIBs falls far short of their theoretical potential. Particularly in extreme scenarios such as high-latitude regions, high-altitude areas, aerospace, polar exploration, and electric vehicles in cold climates, issues such as drastic capacity drop, difficulty in starting, and severe cycle life degradation severely restrict their practical application [5].

**Figure 1:**
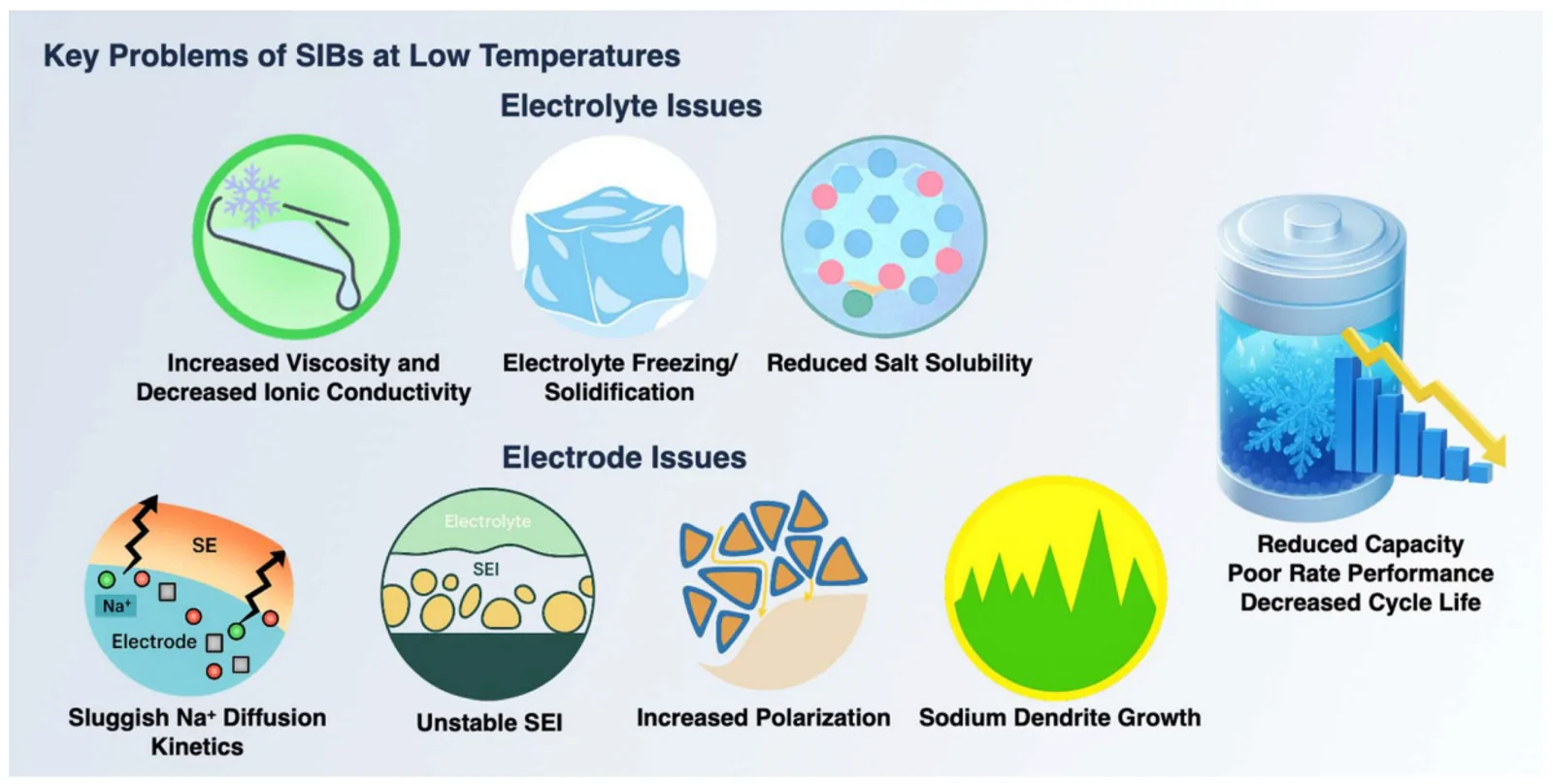
Main challenges for low-temperature SIBs [2].

Elucidating the intrinsic mechanisms of performance degradation in SIBs at LTs and establishing systematic optimization strategies are key to advancing this technology toward practical application. Figure 1 shows the main challenges for LT SIBs [2]. Existing research indicates that the performance degradation of SIBs at LTs results from a multi-factor coupling: an exponential increase in electrolyte viscosity directly reduces ionic conductivity; the kinetic energy barrier for Na^+^ desolvation increases significantly; cathode materials undergo lattice distortion and detrimental phase transitions; and the solid-electrolyte interphase (SEI) on the anode thickens and becomes structurally unstable, leading to a sharp increase in interfacial impedance [6,7]. Therefore, improving a single component often fails to achieve the desired effect, and a systematic approach integrating electrolytes, electrode materials, and interfaces is necessary.

In recent years, research on LT SIBs has grown explosively, yielding numerous illuminating theoretical breakthroughs and technical solutions. This paper aims to systematically review the latest research progress in this field, focusing on core strategies such as electrolyte engineering, electrode material modification, and interface regulation. It delves into the synergistic mechanisms between solvation structure and interfacial chemistry, and, combined with the latest applications of advanced characterization methods and theoretical simulations, distills key principles for the rational design of high-performance LT SIBs. Finally, this review offers forward-looking thoughts and prospects for future research directions, providing a useful reference for researchers in the field.

## 2. Analysis of LT Performance Degradation Mechanisms

The system consists of a cathode, an anode, an electrolyte, and two current collectors placed at the outer layers. Both current collectors are made of aluminum foil (Al foil). Unlike conventional lithium-ion batteries, which require copper foil for the anode current collector, SIBs can utilize aluminum foil on both the cathode and anode sides. This design leverages the compatibility of aluminum with sodium-based electrochemistry, avoids the cost and weight penalties of copper, and contributes to the overall economic and sustainability advantages of SIBs, particularly for large-scale energy storage applications.

### 2.1. Electrolyte Transport Kinetic Limitations

**Figure 2:**
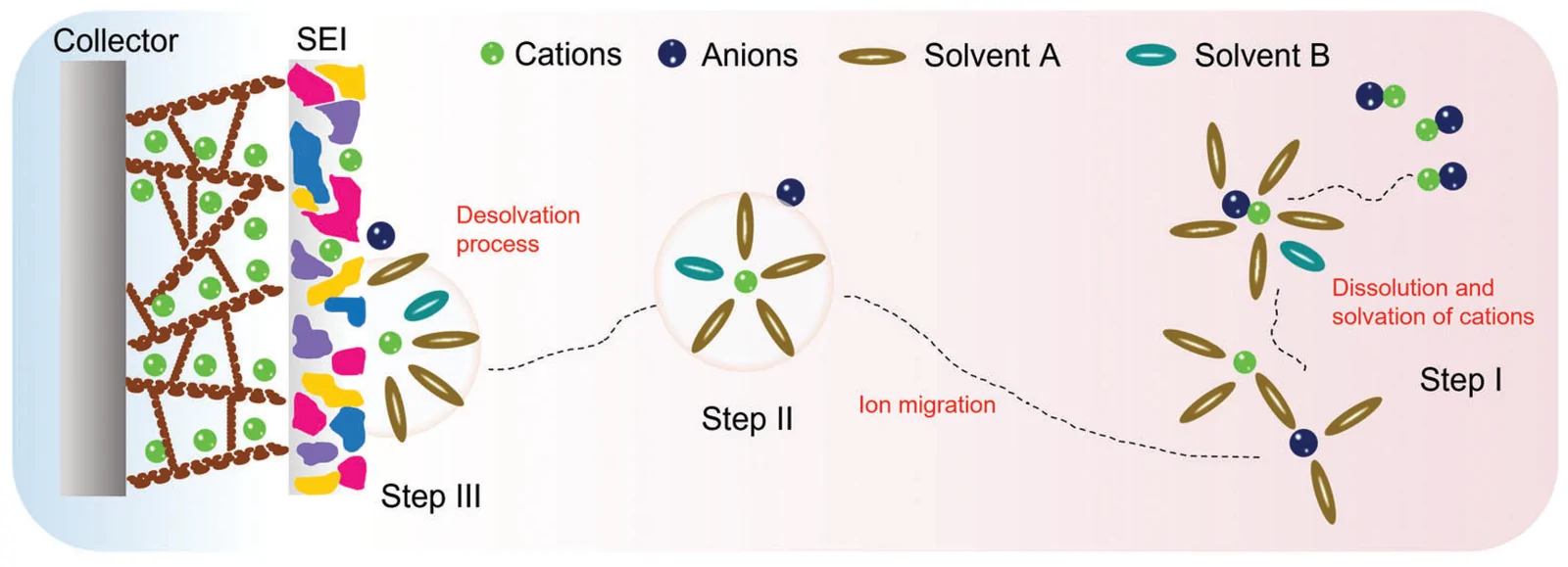
The core understanding of desolvation [11].

The electrolyte, as the medium for ion transport, is the primary factor limiting the performance of SIBs at LTs. Taking the charging process as an example, the transport of Na^+^ in the electrolyte proceeds via three successive steps: (1) desodiated Na^+^ from the cathode coordinates with organic solvent ligands to form solvated Na^+^ complexes (step I); (2) diffusion of solvated Na^+^ within the bulk electrolyte (step II); (3) desolvation prior to intercalation (step III). The physicochemical properties of the electrolyte dominate the overall battery performance throughout these procedures. As the temperature decreases, electrolyte viscosity increases exponentially, the ion transference number drops, and the ionic conductivity of the electrolyte decreases significantly [8]. Meanwhile, the solvation interaction between Na^+^ and solvent molecules is intensified at LTs, which not only increases the migration resistance of Na^+^ in the electrolyte but also raises the desolvation energy barrier at the electrode–electrolyte interface, as shown in Figure 2, making it difficult for Na^+^ to overcome this thermodynamic barrier at the electrode–electrolyte interface and intercalate into the electrode material’s lattice. This kinetic hysteresis manifests as poor rate capability and LT capacity retention at the half-cell level, and as significantly reduced energy output and power density at the full-cell level [9,10].

Notably, the challenges faced by electrolytes at LTs are not limited to the deterioration of physical properties but also include decreased chemical stability. At LT, the decomposition reaction pathways of certain solvent molecules may change, leading to the formation of unstable interfacial products that further exacerbate the increase in interfacial impedance [12]. Therefore, optimizing electrolyte systems requires considering multiple indicators such as freezing point, viscosity, ionic conductivity, desolvation energy, and electrode interface stability, posing more stringent requirements for the design of LT electrolytes.

### 2.2. Lattice Distortion and Phase Transition Instability of Cathode Materials

The electrochemical behavior of cathode materials at LTs directly determines the stability of battery capacity output. SIBs include layered oxides, polyanionic compounds, and Prussian blue analogs (PBAs).

Layered transition metal oxides (e.g., O3-type and P2-type materials) have attracted much attention due to their high theoretical specific capacity. However, during LT electrochemical reactions, Na^+^ intercalation/deintercalation is often accompanied by complex structural evolution and multi-step phase transitions. While these phase transitions are reversible at room temperature, the significantly slowed Na^+^ diffusion kinetics at LTs make these phase transitions incomplete or irreversible, leading to the accumulation of lattice distortion and structural collapse. For example, P2-type Ni-Mn-based layered cathode materials undergo an irreversible P2 → O2 phase transition during charge/discharge, which is particularly pronounced at LTs, severely shortening the cycle life of the material [13].

In addition, interfacial side reactions between layered oxide cathodes and the electrolyte are aggravated under LT conditions. The resultant cathode-electrolyte interphase (CEI) film exhibits prominent drawbacks including uneven thickness, loose structure and poor ionic conductivity, which hinders the normal transport of Na^+^ and elevates the interfacial charge transfer resistance. More severely, this circumstance accelerates the dissolution of transition metal ions. The dissolved metal ions migrate toward the anode surface, destroy the structural stability of the SEI film, and further aggravate the overall capacity degradation of the battery. This serves as a critical inducement for the deteriorated LT cycling performance of layered metal oxide cathodes.

Polyanionic compounds (e.g., Na_3_V_2_(PO_4_)_3_, NaFePO_4_, etc.) feature a 3-D framework structure that provides stable diffusion channels for Na^+^. However, the large-sized anionic groups (PO_4_^3−^, SO_4_^2−^, etc.) of these materials limit their intrinsic electronic conductivity, resulting in low specific capacity. The conductivity further decreases at LTs, which substantially raises the electron migration resistance inside the material, accompanied by increased polarization overpotential and fading of available capacity. PBAs are regarded as promising cathode materials owing to their low production cost and high theoretical capacity. Nevertheless, their practical application at LTs is mainly hindered by the difficult synthesis of high-purity products as well as structural defects and crystal water inside the crystal lattice. A certain amount of crystal water commonly exists in the open framework of PBAs. During LT charge–discharge cycling, such crystal water gradually occupies the diffusion channels and active sites for Na^+^ transportation, triggering irreversible lattice distortion and even structural phase transition, thereby leading to severe capacity degradation under LT conditions.

### 2.3. Anode Interface SEI Instability and Sodium Dendrite Growth

The anode is a critical node in the Na^+^ intercalation pathway, and its LT behavior is decisive for the overall LT performance of the battery. Hard carbon (HC) is currently the most widely studied and commercially promising anode material for SIBs, favored for its suitable plateau potential, good structural stability, and high reversible capacity. However, hard carbon anodes face multiple challenges at LTs. First, the desolvation process consumes most of the energy for ion transport, and the larger ionic radius of Na^+^ means that overcoming the energy barrier at the anode interface requires higher activation energy. Second, the quality of SEI formation strongly depends on the morphology and chemical composition of the anode surface. The defect-rich microstructure of the hard carbon surface often leads to an inhomogeneous SEI layer rich in organic components but deficient in inorganic components, which exhibits high Na^+^ diffusion impedance at LTs. More critically, the sluggish Na+ transport kinetics of HC at LTs and the sodium storage mechanism relying on sodium cluster deposition within pores greatly elevate the risk of sodium dendrite growth. The growth of sodium dendrites may not only trigger internal short circuits and thermal runaway inside the cell, but also pierce the separator to induce severe safety accidents, posing prominent safety hazards to the battery system [14].

Sodium dendrite growth becomes particularly prominent for sodium metal anodes under LT conditions. LT exacerbate the inhomogeneous distribution of ion flux, promoting preferential dendrite growth, while the unstable SEI layer fails to effectively suppress this process. The growth of sodium dendrites can not only lead to internal short circuits and thermal runaway but can also penetrate the separator, causing serious safety accidents.

The degradation mechanism of the SEI film at LTs can be analyzed from three aspects. First, compositional evolution of the SEI film leads to deteriorated ionic conductivity. The SEI film consists of inorganic and organic components; generally, a higher proportion of inorganic components corresponds to superior ionic conductivity. At LT, the reduction and decomposition pathway of electrolyte solvents alters, changing the compositional constitution of the SEI film and reducing the fraction of inorganic phases, which consequently increases the ionic transport resistance of the SEI layer. Second, structural evolution weakens the mechanical stability of the SEI film. The SEI generated under cryogenic conditions exhibits markedly degraded structural compactness, accompanied by abundant internal pores, cracks and structurally fragile domains. Such defects directly reduce the mechanical robustness of the SEI film, disabling its capacity to accommodate volume variation in the HC anode during charge–discharge cycling. Moreover, these structural defects continuously propagate and aggravate upon prolonged cycling, even triggering partial rupture of the SEI layer. The barrier effect of SEI is accordingly destroyed, inducing continuous electrolyte decomposition at cracked sites, irreversible consumption of limited active sodium, excessive SEI thickening, and continuous elevation of interfacial impedance. Third, sluggish formation kinetics exacerbates the inhomogeneity of the SEI film. LT conditions reduce the solubility of sodium salt in the electrolyte and slow down ionic migration kinetics. The non-uniform growth rate of the SEI film over different regions of the electrode results in uneven SEI thickness, further impairing the structural stability of the interfacial passivation layer.

### 2.4. Multi-Factor Coupled Failure Mechanisms

LT performance degradation in SIBs is indeed a typical “multi-factor coupled failure mechanism” process. A detailed discussion of the failure mechanisms in four aspects—electrolyte, cathode, anode, and interfaces—is provided below [2,3,9,15,16,17,18,19,20].

1. The most direct effects of LT on the electrolyte are a sharp increase in viscosity and a drastic drop in ionic conductivity. The viscosity of organic electrolytes increases significantly at LTs, directly restricting the migration rate of Na^+^ in the bulk electrolyte and causing severely sluggish ionic transport kinetics. More critically, the increase in electrolyte viscosity is closely related to the Na^+^ desolvation process. At LT, the binding energy between Na^+^ and solvent molecules intensifies, substantially raising the desolvation energy barrier at the electrode/electrolyte interface. Furthermore, some electrolyte systems may approach or reach their freezing point below −20 °C, leading to localized precipitation or “salting out” of the electrolyte, which triggers catastrophic battery failure.
2. The structural degradation of cathode materials at LTs is equally non-negligible. O3-type layered oxide cathodes face triple structural challenges under LT conditions: structural distortion arising from the mismatch between sodium-ion radius and lattice dimensions, irreversible phase transitions that cause capacity fading, and a sharp increase in the Na^+^ diffusion energy barrier within the bulk. Specifically, the diffusion coefficient of Na^+^ in the cathode bulk decreases significantly at LTs. After Na^+^ enters the cathode lattice from the electrolyte, its migration pathway is hindered by lattice contraction and local strain. This diffusion obstruction further exacerbates the concentration gradient of Na^+^ inside the cathode particles, inducing local lattice distortion. As cycling proceeds, the accumulated distortion may trigger irreversible phase transitions—for example, incomplete transformation from the O3 phase to the P3 phase or hindered reverse transformation—resulting in irreversible loss of active Na^+^ and capacity decay. It is worth noting that the high-entropy strategy has been proven to alleviate this issue to some extent. High-entropy layered oxide cathodes, through multi-element synergy, can effectively suppress local lattice distortion and internal stress while broadening the P3 phase region to enhance Na^+^ transport capability. This corroborates that cathode structural degradation is a core link in LT failure.
3. The failure mechanisms at the anode/electrolyte interface under LT have garnered increasing attention in recent years. HC, the most commonly used anode material for SIBs, experiences LT failure primarily dominated by interfacial degradation rather than bulk damage. At −20 °C, LT not only reduces the ionic conductivity of the electrolyte but also significantly increases the interfacial charge-transfer impedance. The overall sluggish kinetics further induce two mutually reinforcing processes: sodium dendrite growth and continuous thickening of the SEI. These two processes jointly accelerate the consumption of active sodium and the accumulation of interfacial impedance, ultimately leading to irreversible capacity fading. More critically, SEI thickening is not a mere physical process. At LT, the SEI composition tends to be inhomogeneous, and the interaction between Na^+^ and SEI components weakens, further exacerbating interfacial impedance. Meanwhile, dynamic SEI reconstruction causes the interfacial impedance to rise continuously with cycling. This feedback loop—“LT leads to sluggish kinetics → induces dendrite growth and SEI thickening → further increases impedance → exacerbates sluggish kinetics”—constitutes a classic positive-feedback failure circuit. Under extreme temperatures, this interfacial failure mechanism may even evolve into a vicious cycle of “aggravated side reactions → SEI crystallization → impedance increase → further side reactions.”

The failures in the above components do not occur in isolation but are mutually exacerbated through multiple coupled pathways:

Pathway 1: Electrolyte → Cathode coupling. The increase in electrolyte viscosity not only reduces bulk ionic conductivity but also makes Na^+^ desolvation at the cathode/electrolyte interface more difficult. Hindered desolvation means a reduced flux of Na^+^ entering the cathode lattice, which further aggravates the non-uniform Na^+^ concentration and lattice distortion inside the cathode.

Pathway 2: Electrolyte → Anode interface coupling. The decrease in electrolyte ionic conductivity and the rise in interfacial charge-transfer impedance at LTs are mutually causal. The rising impedance drives continuous SEI thickening, which in turn consumes electrolyte components and further increases impedance.

Pathway 3: Cathode ↔ Anode indirect coupling. Structural degradation and hindered Na^+^ diffusion in the cathode reduce the overall utilization efficiency of the Na^+^ inventory in the battery; meanwhile, the persistent accumulation of anode interfacial impedance further restricts Na^+^ intercalation/deintercalation kinetics. The two restrict each other through the “Na^+^ transport chain”—deterioration at either link amplifies the failure degree of the other.

**Figure 3:**
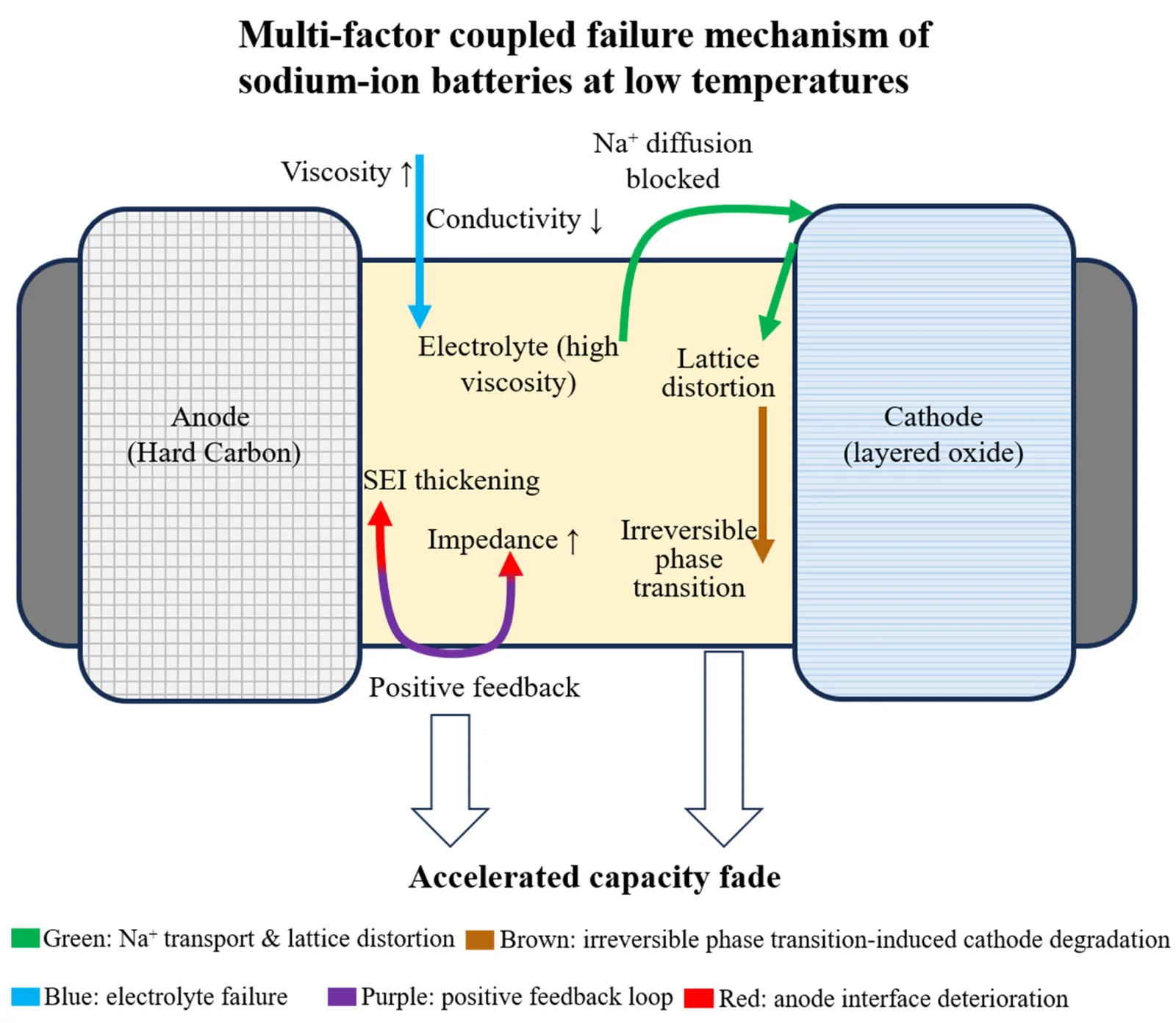
Panorama of multi factor coupled failure mechanisms.

The panorama of multi-factor coupled failure mechanisms is shown in Figure 3, which, based on a battery cross-sectional view, uses arrows of different colors to mark the coupled pathways among increased electrolyte viscosity → decreased ionic conductivity → hindered cathode diffusion/lattice distortion and anode SEI thickening/impedance rise, visually illustrating the positive-feedback loop of “low-temperature cycling—interface degradation—capacity fading.”

**Figure 4:**
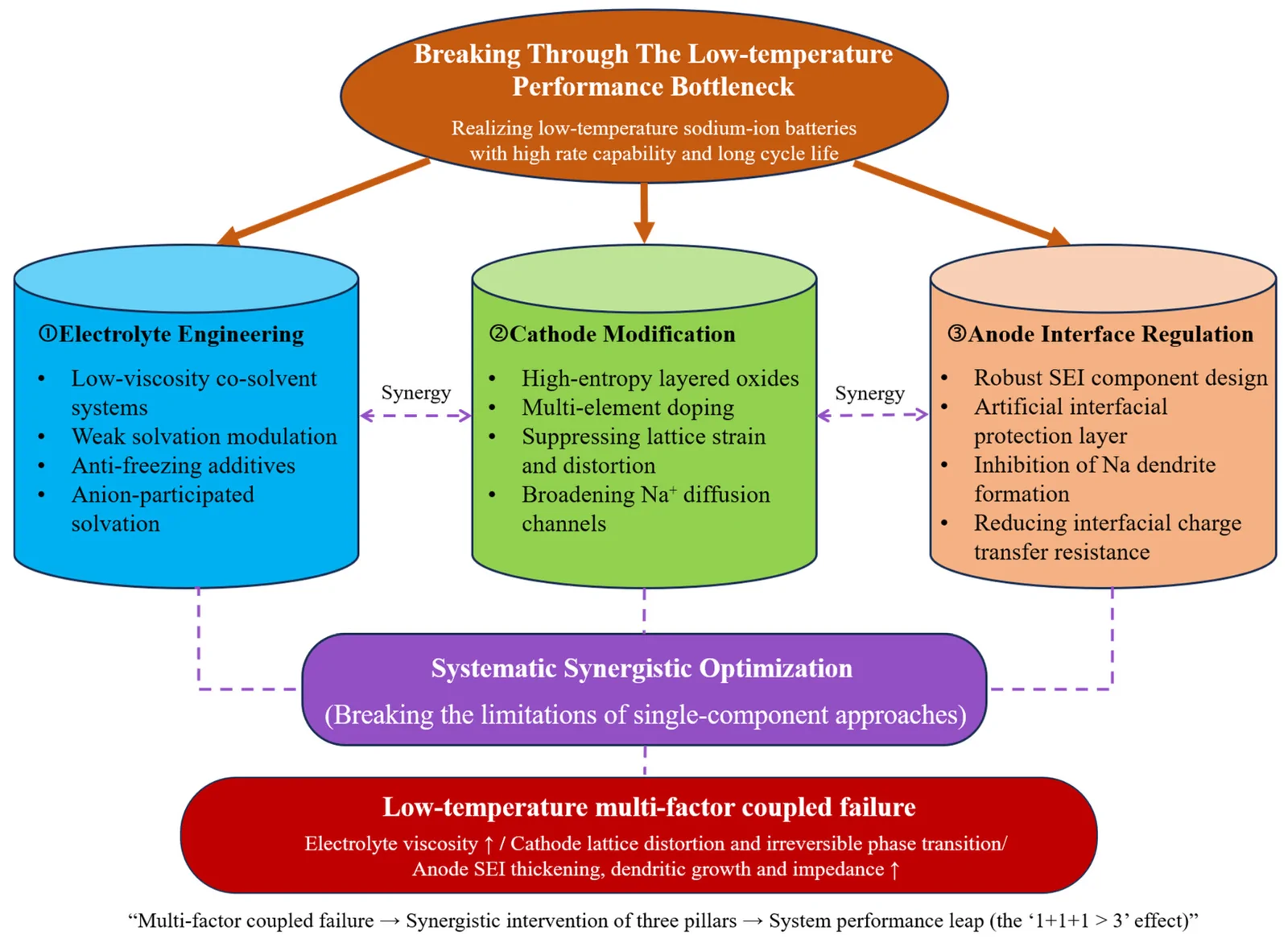
Overview of synergistic optimization strategies.

The above multi-factor coupled failure characteristics clearly indicate that optimizing a single component locally cannot fundamentally solve the problem. Electrolyte engineering, cathode modification, and anode interface regulation must be advanced synergistically. Several emerging strategies in recent years have already embodied this synergistic optimization concept: for example, using a single-molecule additive that integrates sodium compensation, co-solvent, and diluent functionalities to simultaneously address the triple bottlenecks of active sodium loss, electrolyte freezing, and viscosity surge; regulating anion participation to achieve synergistic optimization of LT kinetics and interfacial stability; and through the synergistic design of solvation structure and interface engineering, breaking the long-standing trade-off between ion desolvation and diffusion at LTs. These advances demonstrate that only by treating the electrolyte, cathode, anode, and interfaces as an interconnected holistic system and establishing systematic synergistic optimization strategies can the LT performance bottleneck of sodium-ion batteries be effectively overcome. An overview of synergistic optimization strategies is presented in Figure 4, with “electrolyte engineering—cathode modification—interface regulation” as the three pillars, illustrating the overall framework of systematic synergistic optimization.

## 3. Electrolyte Engineering Strategies

Electrolytes are mainly composed of sodium salts, organic solvents and functional additives. To optimize the LT performance of electrolytes, conventional strategies focus on regulating physical properties via compositional modulation, such as screening solvents with low freezing points and sodium salts with high dissociation capacity to improve the ionic conductivity of electrolytes. With in-depth research in recent years, it has been verified that modulating the solvation structure of Na^+^ can reduce the desolvation energy barrier of Na^+^ and the energy barrier for Na^+^ penetrating the SEI film, accelerate the interfacial transport kinetics of Na^+^, and thus effectively boost the LT performance of SIBs. Typical implementation approaches include constructing weakly solvated electrolytes, eliminating the Na^+^ desolvation process, and regulating the solvation configuration via functional additives.

### 3.1. Design of Low-Freezing-Point Solvent Systems

The solvent component in the electrolyte plays a decisive role in LT ion transport behavior. Common solvents applied in SIBs mainly include esters (e.g., Propylene Carbonate, Ethylene Carbonate, Dimethyl Carbonate, Diethyl Carbonate, Ethyl Methyl Carbonate) and ethers (Dimethoxyethane, Tetrahydrofuran). Ester-based solvents possess favorable chemical stability and can facilitate the formation of robust SEI films on electrode surfaces, yet they suffer from high freezing points and viscosity, leading to inferior LT performance [21]. Mixing two or three types of solvents to formulate binary or ternary electrolytes can ameliorate LT performance to a certain extent. Nevertheless, this strategy fails to break through the interfacial kinetic bottleneck induced by strong solvation effects.

**Figure 5:**
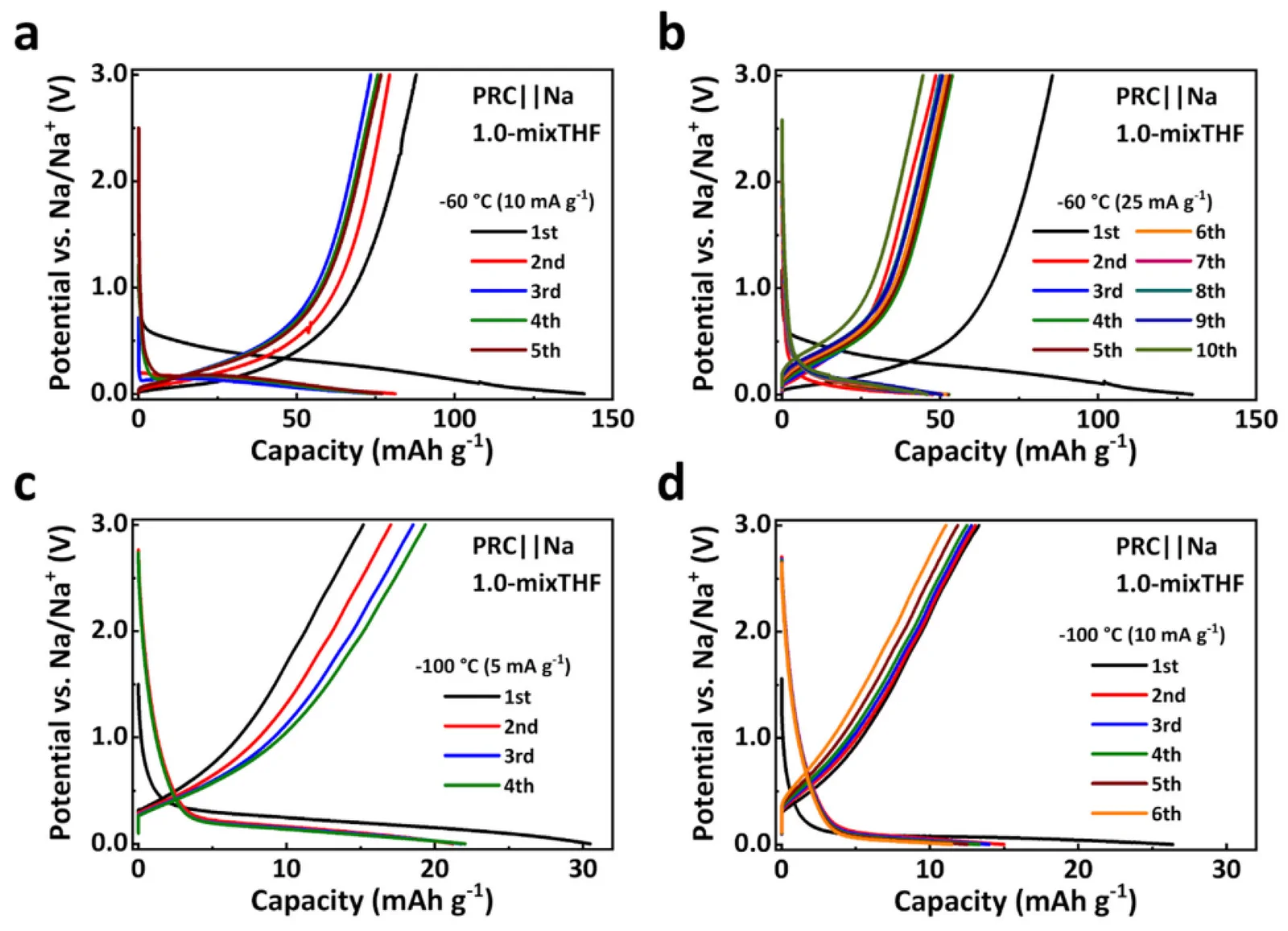
Low-temperature electrochemical performance of PRC||Na cells in 1.0-mixTHF. Galvanostatic voltage profiles at (**a**) −60 °C and 10 mA g^−1^, (**b**) −60 °C and 25 mA g^−1^, (**c**) −100 °C and 5 mA g^−1^, and (**d**) −100 °C and 10 mA g^−1^ [23].

Ether solvents have shown significant advantages in ultra-low-temperature SIBs research due to their low freezing points and moderate viscosities [4,22]. Soohwan Kim et al. systematically studied the solvation behavior of NaPF_6_ in THF/2-MeTHF mixed solvent systems, finding that the fine-tuning of the solvation structure could be achieved by adjusting the solvent ratio, allowing precise control over the freezing point, solubility, and Na^+^ solvation structure. The optimized mixed-solvent electrolyte promoted extensive anion participation, forming a stable and inorganic-rich SEI layer, thereby achieving low overpotential and uniform Na^+^ deposition. Based on this strategy, the developed electrolyte enabled SIBs to achieve reversible capacities of 81 mAh g^−1^ and 21 mAh g^−1^ at −60 °C and −100 °C, respectively, breaking the limit of ultra-low-temperature operation [23]. As shown in Figure 5, based on the exceptional stability of the 1.0-mixTHF as an SIB electrolyte and the low freezing point of the solvents, we explored the viability of the PRC||Na cell in the 1.0-mixTHF for extremely cold environments. Figure 5a demonstrates that at −60 °C, the PRC||Na cell delivers 81.2 mAh g^−1^ at 10 mA g^−1^ and shows rechargeability for 5 cycles. Figure 5b shows that the PRC||Na cell also operates with higher current densities of 25 mA g^−1^, exhibiting > 50.0 mAh g^−1^ rechargeable capacities. Figure 5c,d show that even at −100 °C, the PRC||Na cell achieves >20 mAh g^−1^ at 5 mA g^−1^ and >13.0 mAh g^−1^ at 10 mA g^−1^ for reversible capacities. Carboxylate ester solvents (e.g., methyl butyrate, ethyl acetate) are also widely studied due to their low freezing points and high dielectric constants, and are considered promising solvent types for LT SIBs [24].

### 3.2. Weakly Solvating Electrolytes and Desolvation Energy Barrier Regulation

The desolvation process is the primary energy dissipation step for Na^+^ migration at the electrode–electrolyte interface [25]. At low temperatures, the strong coordination between solvent molecules and Na^+^ further increases the desolvation energy barrier, making it the dominant factor limiting LT electrochemical kinetics [26,27]. The design concept of weakly solvating electrolytes (WSEs) is precisely to reduce the desolvation energy barrier by weakening the coordination strength between Na^+^ and solvent molecules, thereby accelerating interfacial Na^+^ transport [24,28,29]. Practical implementation strategies include competitive coordination with co-solvents featuring low donor number (DN), anion modulation, and solvation reconstruction via localized high-concentration electrolyte design.

low-DN weakly coordinating solvent engineering represents the most classic molecular design strategy for constructing weakly solvated electrolytes and tuning the Na^+^ desolvation energy barrier. In this strategy, low-DN solvents (e.g., 2-Methyltetrahydrofuran, Cyclopentyl methyl ether, Dimethoxymethane, methyl propionate, methyl formate) are adopted as co-solvents or primary solvents to reconstruct the primary solvation sheath of Na^+^ via competitive coordination, so as to reduce the desolvation energy barrier. However, traditional WSEs face a fundamental paradox: the solvation structure is highly sensitive to temperature fluctuations. As the temperature decreases, the coordination between Na^+^ and solvent molecules may strengthen due to reduced thermal motion, causing the original “weak solvation” characteristics to fail at low temperatures [30]. To address this limitation, Zhenxin Huang et al. proposed a breakthrough strategy of solvent-solvent interaction regulation. By leveraging interactions between solvent molecules to effectively fuse strong and weak solvents, they constructed a temperature-robust solvation structure [27]. The single-component formulation of WSEs promotes the formation of highly localized ordered structures within the electrolyte, which to some extent impedes Na^+^ transport kinetics and reduces ionic conductivity. In this work, they designed an LT electrolyte for sodium-metal batteries by combining a weakly solvating solvent with a strongly solvating one, leveraging the intermolecular interactions between the solvent molecules to construct a temperature-robust solvation structure. This strategic mixing increases the diversity of solvation structures and mitigates local ordering, ultimately yielding an electrolyte that exhibits higher ionic conductivity and a lower freezing point than single-solvent systems. This design allows the continuous participation of anions in the Na^+^ solvation sheath even under significant temperature fluctuations, stabilizing the desolvation behavior. Based on this strategy, the developed electrolyte extended the operating temperature range of sodium batteries to −40–45 °C.

Similarly, Yiwen Gao et al. utilized the hydrogen-bonding interaction between dimethyl sulfite (DMS) and glutaronitrile (GN) solvents to regulate the Na^+^ solvation structure and effectively improve the oxidative stability of the single DMS solvent. The hydrogen bond is formed between the electron-deficient δH^+^ (on GN) and the electron-rich δO^−^ (on DMS) of the two solvents. This hydrogen-bonding interaction not only enhances the antioxidant capability of DMS molecules but also induces the reorganization of the Na^+^ solvation structure, resulting in an anion-rich and loosely coordinated solvation sheath. Such a loose solvation structure effectively lowers the desolvation energy barrier of Na^+^, thereby facilitating faster Na^+^ transport and desolvation processes at the electrode/electrolyte interface; this rapid kinetic behavior contributes to improving the battery performance over a wide temperature range. Benefiting from this, the assembled NaNi_0.33_Fe_0.33_Mn_0.33_O_2_ (NFM)||Na half-cell successfully achieved stable operation within a broad temperature window from −55 to 60 °C [31].

Solvation modulation can also be realized by introducing strongly coordinating anions into conventional electrolytes. For instance, hybrid sodium salt systems dissolve two types of sodium salts in a single solvent medium to supply two kinds of anions. One sodium salt serves as the fundamental salt to guarantee satisfactory ionic conductivity, while the other provides strongly coordinating anions to participate in solvation regulation and reduce the activation energy for desolvation.

High-concentration electrolytes (HCEs) can effectively reduce the desolvation energy barrier, yet they suffer from inherent drawbacks including excessively high viscosity, suppressed ion transport, and severe sodium salt precipitation at LTs. Localized high-concentration electrolytes (LHCEs) employ inert fluorinated diluents to reduce the bulk viscosity while preserving the microstructural features of high-concentration solvation clusters. Benefiting from the merits of low viscosity in conventional dilute electrolytes and optimized solvation sheath structure in high-concentration electrolytes simultaneously, LHCEs are regarded as a promising candidate for LT SIBs.

### 3.3. Functional Additives and Cooperative Solvation Structure Construction

In addition to the choice of main solvent and sodium salt, functional electrolyte additives play an irreplaceable role in regulating interfacial chemistry and stabilizing electrode–electrolyte interfaces [32,33,34]. Although additives are used in small amounts, their impact on the composition and structure of the SEI/CEI is often significant [35,36,37]. Additives can be categorized by their functions into film-forming additives, low-freezing-point additives, additives for solvation structure optimization, and other types.

**Figure 6:**
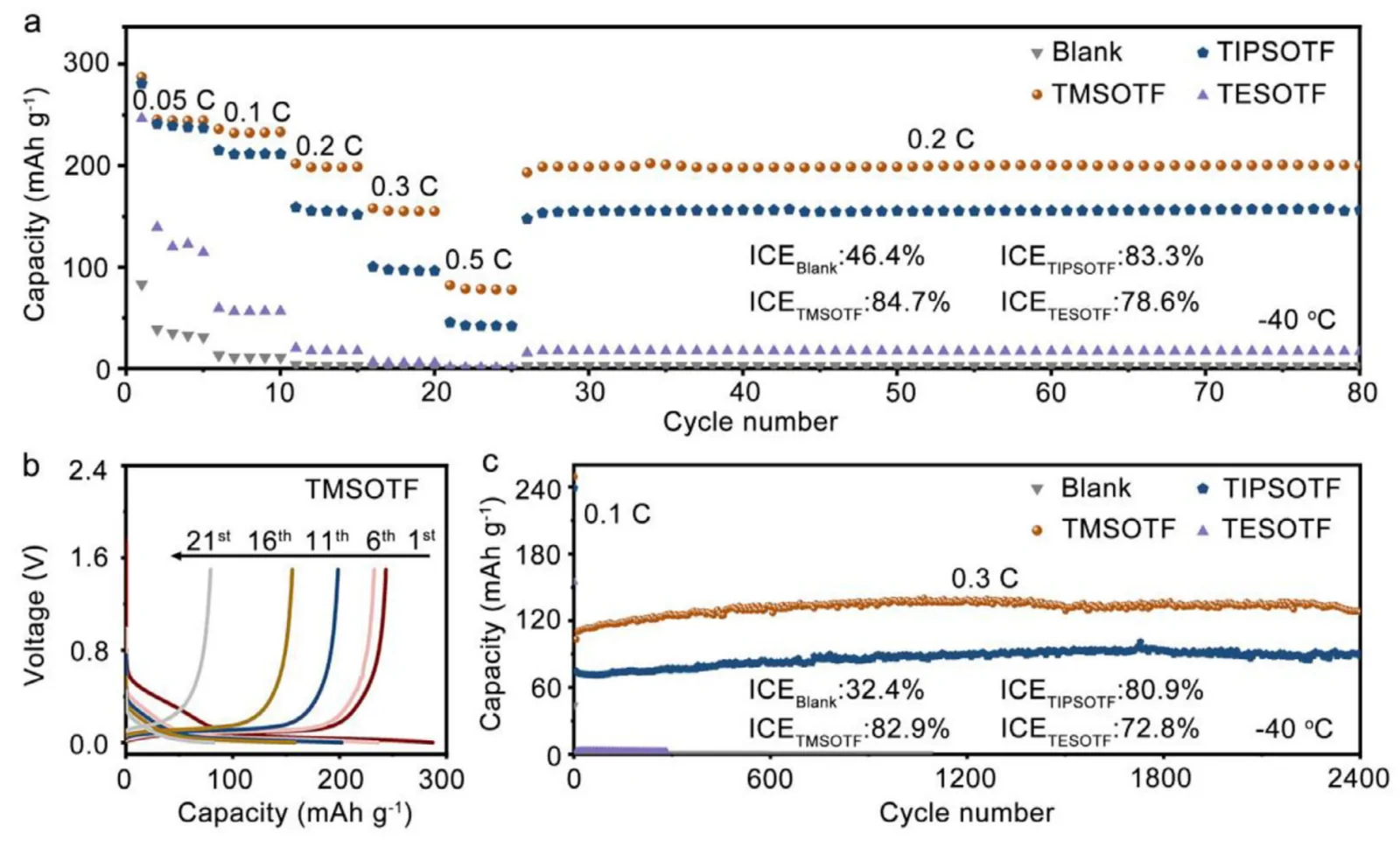
Electrochemical performance of Na||HC cells with different electrolytes at −40 °C. (**a**) Rate capability of the Na||HC cells (0.05 C, 0.1 C, 0.2 C, 0.3 C, 0.5 C each cycled for five cycles, followed by long-term cycling at 0.2 C). (**b**) Charge–discharge curves of Na||HC cells with TMSOTF at different cycles at −40 °C. (**c**) Cycling performance of Na||HC cells with different electrolytes at −40 °C [38].

The core function of film-forming additives lies in preferential oxidative/reductive decomposition at the electrode interface to participate in the construction of stable, low-impedance SEI/CEI films. Such interfacial layers can continuously suppress the sustained decomposition of solvent molecules and dendrite growth under LT operating conditions, serving as a critical technical approach to improve the LT cycling stability of batteries. Jiale Zheng et al. recently revealed a critical interfacial mechanism: at low temperatures, linear solvent molecules tend to aggregate within the inner Helmholtz plane (IHP), forming a solvent-derived SEI with sluggish Na^+^ diffusion kinetics [38]. To address this, they proposed using trimethylsilyl trifluoromethanesulfonate (TMSOTF) as an electric double layer regulator. TMSOTF generates radicals in situ within the IHP, and the radical-induced polarization interactions with solvent molecule orbital overlap interrupt solvent aggregation, redistributing electron density and reducing molecular polarity. This mechanism significantly weakens intermolecular ordering, promotes interface reconstruction, accelerates mass transport, and induces the formation of an inorganic-rich SEI layer. To evaluate the performance of cells with various electrolytes, they investigated the electrochemical stability of Na||HC cells (Figure 6), with sodium metal and HC as the electrodes. As described in Figure 6a, Na||HC cells with TMSOTF electrolyte exhibited significantly higher charge capacities at current densities of 0.05 C, 0.1 C, 0.2 C, 0.3 C, 0.5 C, and 0.2 C, respectively, compared to those with the TIPSOTF electrolyte, demonstrating the superior rate performance of TMSOTF electrolyte (Figure 6b). Benefiting from interfacial restructuring enabled by the EDL regulator, the TMSOTF electrolyte supported long-term cycling with high reversibility in Na||HC cells at low temperatures. Notably, the Na||HC cell with TMSOTF electrolyte achieved an initial Coulombic efficiency (ICE) of 82.9% at −40 °C, outperforming cells with TIPSOTF, TESOTF, and blank electrolytes (Figure 6c). The TMSOTF-based electrolyte enabled commercial hard carbon anodes to achieve a stable cycle life exceeding 2400 cycles at −40 °C, whereas conventional electrolyte systems could not even maintain normal cycling under the same conditions.

Low-freezing-point additives are designed to further reduce the freezing point of electrolytes and enhance ionic transport efficiency at LTs. At present, commonly adopted low-freezing-point additives mainly include adiponitrile (ADN) and inorganic salts such as CaCl_2_. These additives function by disrupting the directional hydrogen bonding between solvent molecules and inhibiting the formation of ordered crystalline structures at LTs. Accordingly, the freezing point of the electrolyte is decreased without an obvious increase in electrolyte viscosity. Meanwhile, these additives exhibit excellent chemical stability, avoiding parasitic side reactions at the electrode–electrolyte interface and imposing no adverse effects on interfacial stability.

Solvation-structure-regulating additives can directionally modify the solvation configuration of Na^+^ relying on the characteristics of their functional groups and incorporate more anions into the solvation shell, which is defined as the anion anchoring strategy.

Furthermore, anion anchoring strategies have also shown great potential for regulating interfacial chemistry. Bin Qiu et al. reported a method to optimize the microstructure of ether-based electrolytes via anion anchoring, confirming that an OTF^−^ anion-anchored solvation structure facilitates the formation of an inorganic SEI rich in NaF and Na_2_O. The optimized HC||Na half-cell achieved a reversible specific capacity of 137.0 mAh g^−1^ at 5 C and −20 °C, with a capacity retention of 96.57% after 2000 cycles [39].The full cell assembled with a NaFePO_4_ cathode exhibits stable cycling performance even at 0 °C, and the assembled pouch cell can successfully power an LED light, demonstrating its practical application prospects.

## 4. LT Modification Strategies for Cathode Materials

### 4.1. Surface Coating and Metal Ion Doping of Layered Oxides

**Figure 7:**
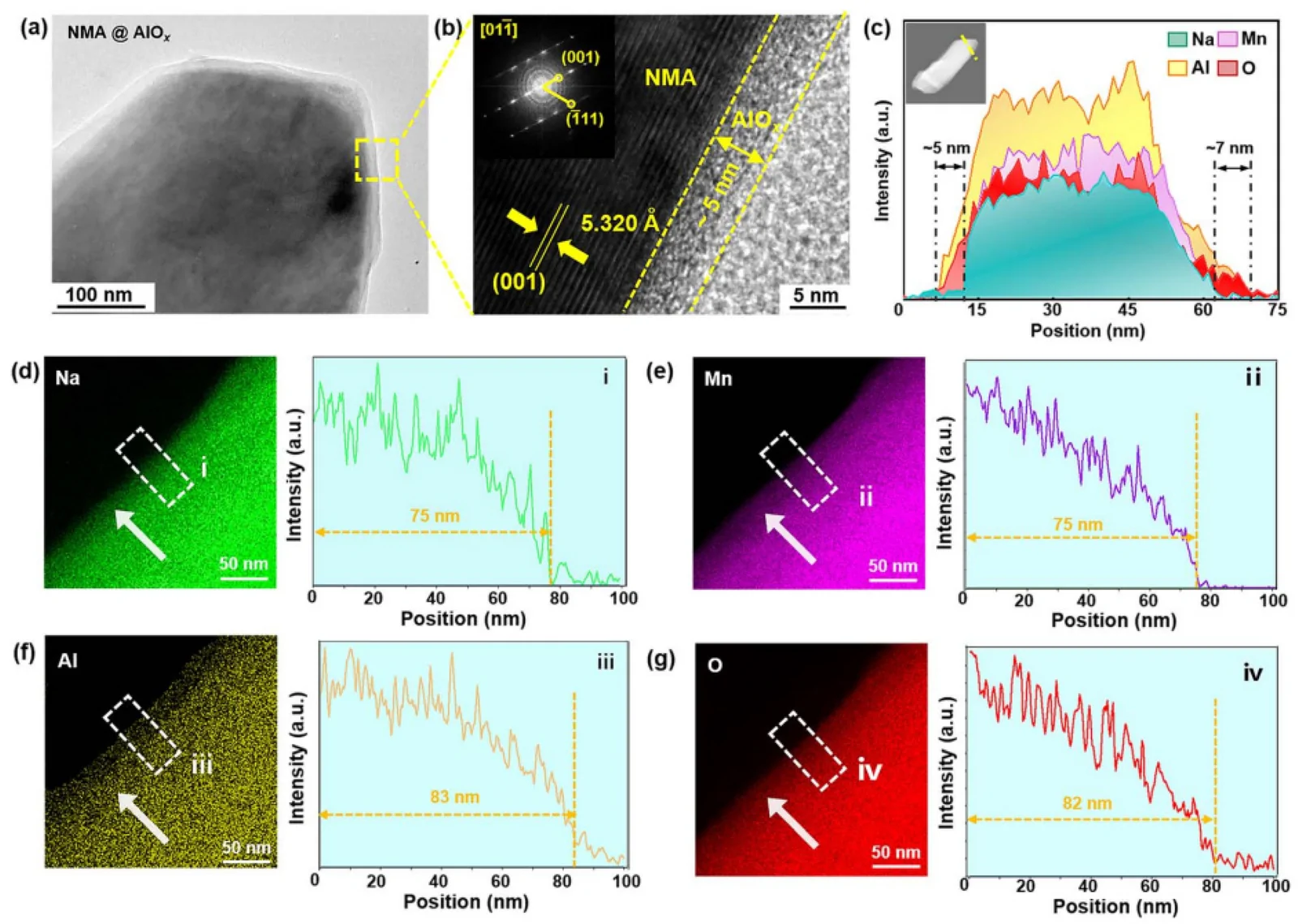
Morphology characterization of the NMA@AlO_x_ sample. (**a**) TEM image of NMA@AlO_x_. (**b**) HRTEM image of NMA@AlO_x_ (inset: FFT image and corresponding Bragg spots). (**c**) STEM line-scan profile of NMA@AlO_x_ cross-section (inset: STEM image of NMA@AlO_x_). (**d**–**g**) EDX mapping of Na (**d**), Mn (**e**), Al (**f**) and O (**g**) elements (**i**–**iv** show the corresponding line-profiles of the marked regions in the EDX images) [43].

Layered transition metal oxides are mainstream cathode materials due to their high specific capacity and good structural tunability [40,41]. However, complex phase transitions and sluggish Na^+^ diffusion kinetics at low temperatures are major bottlenecks [42]. Surface coating is considered one of the most direct strategies to improve interfacial stability. Surface coating refers to depositing one or multiple layers of diverse materials on the surface of cathode materials via physical or chemical routes to endow the materials with new functionalities or upgrade their intrinsic properties. Conductive carbon and other functional materials are widely utilized as coating layers. Conductive carbon coating improves the electronic conductivity of particles and reduces the ohmic resistance at LTs, whereas it fails to effectively isolate parasitic interfacial reactions with the electrolyte, hence it is commonly adopted as an auxiliary composite coating. In contrast, inert oxide coatings construct a physical barrier through compact nanoscale oxide films, which isolate the direct contact between cathode active materials and electrolyte and restrain the continuous growth of interfacial impedance during repeated LT charge–discharge cycles. Research from Zelin Ma et al. showed that AlO_x_-coated O′3-NaMn_0.6_Al_0.4_O_2_ (NMA@AlO_x_) cathode material achieved a capacity retention of 83.2% after 100 cycles at −20 °C, outperforming the uncoated control sample [43]. To clearly observe the coating layer of NMA@AlO_x_, a series of comparative morphological characterization techniques were systematically employed to examine the samples. Figure 7 presents the morphological characterization of the NMA@AlO_x_ samples.

Metal ion doping, which modifies the Na^+^ diffusion channels and electronic structure at the lattice scale, has also achieved notable progress [44,45,46]. Metal ion doping modulates the electronic structure and ionic diffusion pathways of cathode materials by introducing specific ionic dopants to boost their electrochemical performance at LTs. Representative dopants include Ni, Mn, Ti, Nb, and potassium ions (K^+^). Nb doping narrows the electronic bandgap and lowers the energy barrier for ionic diffusion, which remarkably accelerates the rapid transport of electrons and Na^+^ especially under low Na^+^ concentration conditions. Nb-doped P2-Na_0.75_Ni_0.31_Mn_0.67_Nb_0.02_O_2_ cathode material retained 76% capacity after 1800 cycles at −40 °C and a current density of 368 mA/g, attributed to the effective suppression of detrimental P2-to-other phase transitions by Nb doping. K^+^ doping can effectively expand the lattice volume of Na_3_V_2_(PO_4_)_3_(NVP), thereby optimizing the migration pathways for Na^+^. K^+^-doped Na_2_KV_2_(PO_4_)_3_ retained about 72 mAh g^−1^ at −25 °C, a significant improvement over the undoped material. Ti doping alleviates the variation in lattice parameters and phase structure during Na^+^ intercalation/deintercalation, which facilitates the structural integrity of crystals. Ni doping contributes to enhancing the reversible specific capacity of electrodes, while Mn doping suppresses the impedance rise during LT cycling. Nevertheless, the performance improvement achieved by single-element doping is limited. Accordingly, multi-element co-doping and gradient doping strategies have been proposed for further optimization. Although electrochemical performance is moderately enhanced, these modification approaches still face bottlenecks including inhomogeneous microscopic distribution of dopants, poor compatibility with full-cell systems, and unsatisfactory fabrication processes.

It is worth considering that the synergistic application of coating and doping strategies often produces a “1 + 1 > 2” enhancement effect [47,48]. The synergistic effect of nanostructure engineering, surface coating, and metal ion doping is a key pathway to improving the low-temperature performance of cathode materials, and this multi-strategy synergy is expected to effectively mitigate detrimental phase transitions at low temperatures.

### 4.2. Structural Engineering and Carbon Composite of Polyanionic Materials

Polyanionic compounds mainly include phosphates, fluorophosphates, and pyrophosphates. Owing to their rigid 3-D framework and superior thermal stability, these materials deliver distinctive structural advantages for LT applications [49,50]. However, the large anionic groups limit the electronic conductivity and rate capability. Compositing polyanionic materials with conductive carbon is a mainstream strategy to overcome this bottleneck [51,52]. Carbon materials feature easy availability and excellent electrical conductivity. Coating the outer surface of polyanionic compound particles with carbon can not only construct a highly conductive network, but also disperse active material particles and inhibit particle agglomeration/growth. As a result, the specific surface area of active materials is enlarged effectively and the diffusion path of Na^+^ is shortened. Meanwhile, the carbon coating serves as a buffering layer to alleviate volume expansion during charge and discharge, thereby prolonging the cycling lifespan of electrodes. Carbon-coated Na_3_MnZr(PO_4_)_3_@C-rGO composite achieved a discharge capacity of 94.7 mAh g^−1^ at −15 °C and a capacity retention of 79.6% after 1500 cycles, fully demonstrating the effectiveness of the carbon composite strategy in enhancing LT electron transport efficiency. Nevertheless, conventional carbon coating techniques such as solid-state pyrolysis, gas-phase deposition and liquid-phase deposition hardly achieve ultrathin and homogeneous carbon coatings simultaneously. These methods usually require a high carbon loading, which reduces the volumetric energy density of electrode materials and induces uneven coating morphology, further impairing the synergistic transport of ions and electrons. Accordingly, fabricating ultrathin carbon coating layers has become a prominent research focus. Atomic layer deposition (ALD) is theoretically capable of producing atomically uniform thin films, yet it suffers from prohibitive costs, sophisticated equipment and incompatibility with large-scale manufacturing. Hu Anyu et al. synthesized NFPP@C-FAC composites via a precursor-induced quasi-atomic layer deposition (quasi-ALD) strategy, obtaining a uniform carbon layer with an ultralow thickness of only 2.1 nm [53]. Xu Yuhan et al. modified NFPP via carbon coating combined with B doping to enhance the graphitization degree of the carbon shell. Experimental characterizations verify that the boron-induced highly graphitized carbon coating can remarkably accelerate electron and ion transport kinetics [54].

Nevertheless, it should be noted that the energy density of polyanionic materials is inherently limited by their large molecular mass, an intrinsic limitation that cannot be fully overcome by simple structural engineering. Therefore, while pursuing LT cycling stability, balancing energy density with other electrochemical performance indicators is an issue that requires careful consideration in the research of such materials [55].

### 4.3. Morphology Control and Heteroatom Doping of Prussian Blue Analogs

PBAs have attracted widespread attention due to their extremely low cost and high theoretical capacity. However, the synthesis of high-purity PBAs materials at LTs is itself a major challenge [56]. Conventional aqueous liquid-phase synthesis inevitably introduces substantial crystal water and lattice defects. These defects not only block electrochemically active sites but also induce parasitic side reactions that destabilize the electrode–electrolyte interface, thereby severely limiting the LT electrochemical performance of SIBs [57,58]. To address these challenges, Wang Jiangan et al. [58] proposed a nonaqueous synthetic strategy to fabricate PBAs with low crystal water content and suppressed lattice defects. Peng Jian et al. developed an ice-assisted synthetic route to synthesize highly crystalline PBA materials, which achieved remarkable enhancement in the intrinsic electrochemical performance of the electrode material [59]. Jing Zhongxin et al. put forward an acid-assisted synthetic method. The as-fabricated FeVO-PBA cathode exhibited improved crystallinity as well as greatly reduced crystal water and structural defects. The full cell constructed with a FeVO-PBA cathode and HC anode maintained stable operation at −30 °C, verifying its excellent low-temperature electrochemical properties [56].

Microstructure regulation is another effective way to enhance the LT performance of PBAs [60]. Regulation of pore structure, crystal morphology and other microstructural features can shorten the diffusion pathway of Na^+^ and drastically accelerate the Na^+^ diffusion kinetics [61]. Studies have shown that Prussian blue/carbon nanotube composites maintained a capacity of 52 mAh g^−1^ at −25 °C and 6 C, demonstrating the effectiveness of microstructure engineering for LT applications. Furthermore, ion co-doping strategies (e.g., Cs^+^/Zn^2+^ co-doping) have also shown potential for improving the LT electrochemical behavior of PBAs [62].

## 5. LT Optimization Strategies for Anode Materials

### 5.1. Structural Defect Engineering and Pore Design of Hard Carbon

HC is the most commercially promising anode material for SIBs due to its tunable microstructure and superior sodium storage capacity [63,64,65,66]. However, the slow Na^+^ transport kinetics in the plateau region of hard carbon and the sodium storage mode based on sodium cluster deposition pose dual challenges at LTs. Therefore, regulating the pore structure and surface chemistry of hard carbon through defect engineering is a core strategy to enhance its LT kinetic performance [67,68]. The pore structures of HC can be classified into closed micropores, open micropores (<2 nm), mesopores (2–50 nm), and macropores (>50 nm). The optimal pore configuration consists of dominant closed micropores (contributing to plateau capacity for sodium storage), a small proportion of interconnected mesopores (acting as ionic transport channels), and trace macropores (for mechanical stress buffering), while excessive open macropores should be avoided.

Mingxin Song et al. prepared high-performance hard carbon anodes via an LT hydrogen reduction combined with high-temperature carbonization strategy, effectively enhancing the sodium storage kinetics [69]. The morphological features of the final carbon materials were examined by scanning electron microscopy (SEM) and high-resolution transmission electron microscopy (HRTEM). All samples exhibited spherical morphology inherited from corn starch, with particle sizes of approximately 10 μm. Such spherical active materials favored mixing with conductive agents and binders to form a densely packed structure, thus boosting the volumetric energy density of the battery. HRTEM images revealed the microcrystalline structure of the carbon samples, which mainly consisted of highly disordered and quasi-graphitic domains. More importantly, the implementation of a solvent co-intercalation strategy offered a new route to realize rapid ion transport under LT conditions [70].

**Figure 8:**
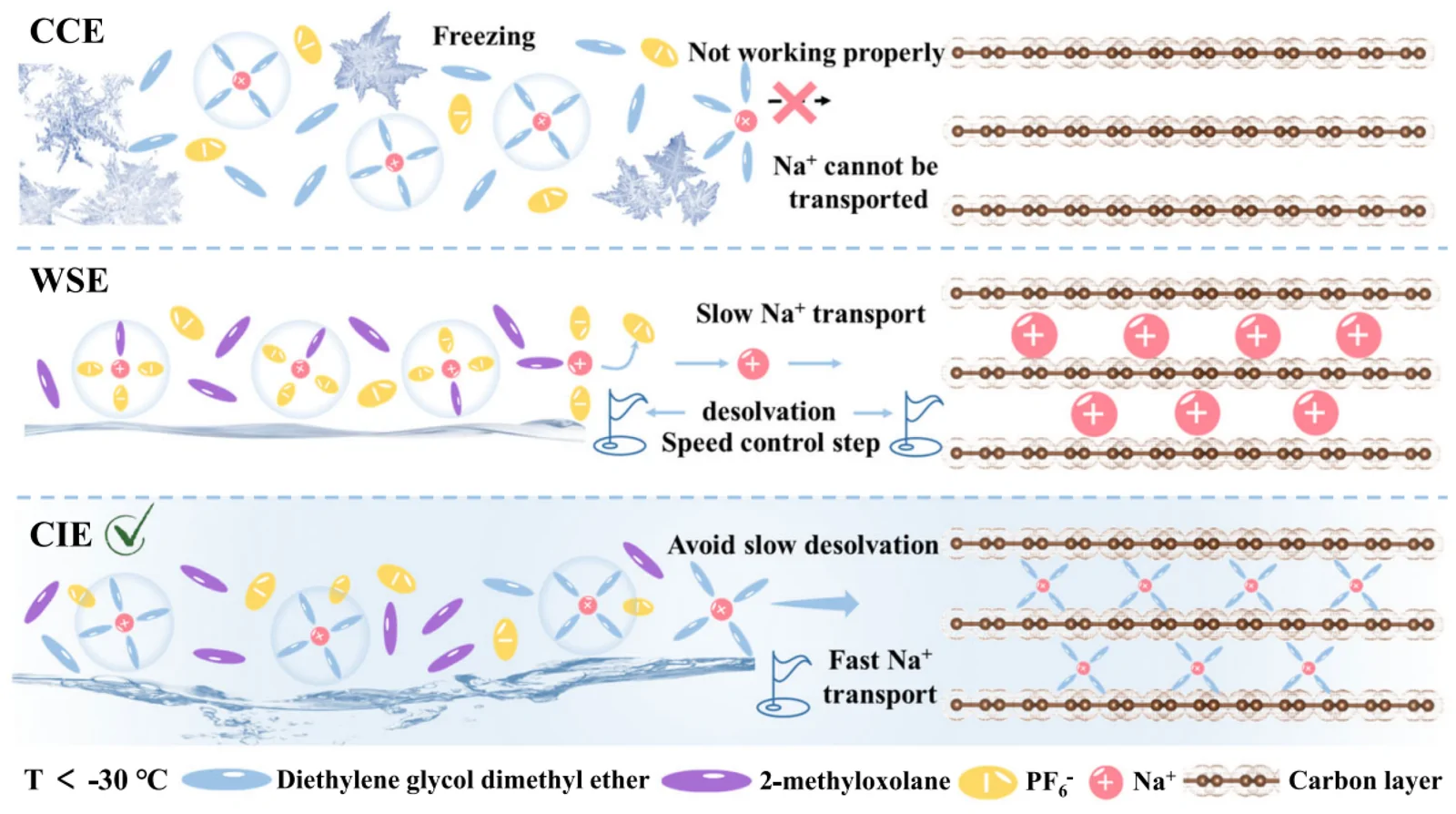
Schematic diagram of ion transport mechanism in various electrolytes at LTs [71].

**Figure 9:**
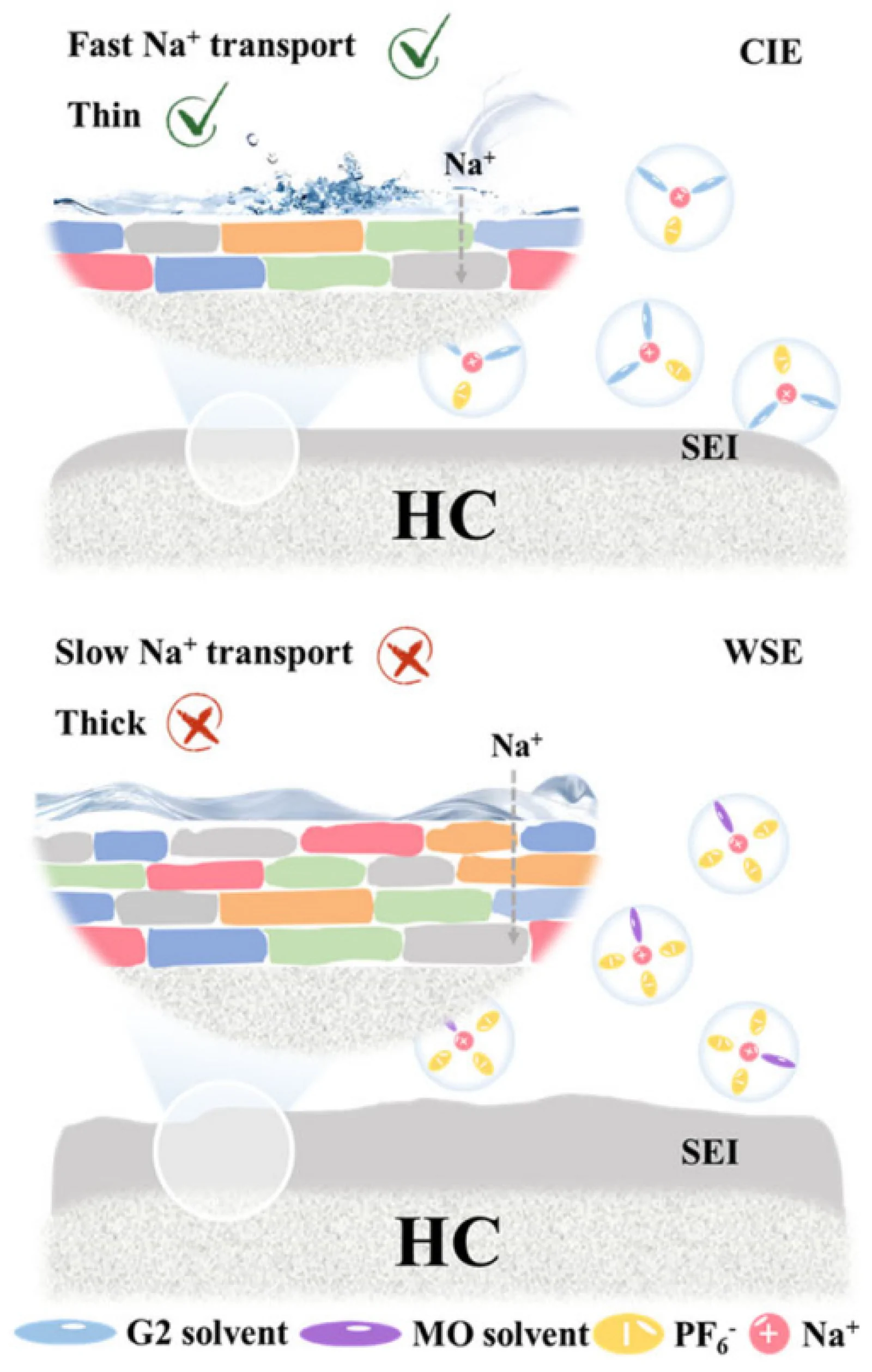
Schematic illustration of the SEI formed in CIE and WSE [71].

Meng Li et al. designed a co-intercalation electrolyte (CIE) tailored for the HC anode, which successfully overcame the trade-off between Na^+^ desolvation and ionic conductivity in the electrolyte. This strategy not only ensures fast ion diffusion in the electrolyte and hard carbon but also optimizes the solvation structure, promoting the formation of a thin and inorganic-rich SEI layer. To highlight the superiority of their designed electrolyte, Meng Li et al. also employed a common co-intercalation ether electrolyte (CCE) and a weakly solvated electrolyte (WSE) as controls. The ion transport mechanisms of the various electrolytes at LTs are illustrated in Figure 8, among which CIE exhibits the fastest ion transport rate due to its co-intercalation behavior. Overall, although WSE forms an inorganic-rich SEI, the excess anions in the solvation sheath lead to an excessively thick SEI, thereby increasing the interfacial resistance. In contrast, CIE can form an inorganic-rich, thin, and dense SEI, effectively facilitating ion transport through the SEI (Figure 9). The prepared hard carbon anode achieved an initial Coulombic efficiency of 80.5% at −50 °C and 20 mA g^−1^, with a capacity retention of 93% after 200 cycles. More impressively, Ah-level pouch full cells based on this strategy achieved a stack-level specific energy of 163 Wh kg^−1^ at 25 °C and maintained 107 Wh kg^−1^ at −50 °C, fully demonstrating the practical feasibility of this strategy [71].

### 5.2. Heteroatom Doping and Electronic Structure Regulation

Heteroatom doping introduces elements such as N, P, S, and B at the edge sites or intrinsic defect sites of the hard carbon lattice, altering the local electron density distribution and thereby optimizing the adsorption energy and migration pathway of Na^+^ [72,73,74,75]. Doping types mainly include substitutional doping, surface functional group doping, and interstitial lattice doping. The effects of single-heteroatom doping are summarized in Table 2.

**Figure 10:**
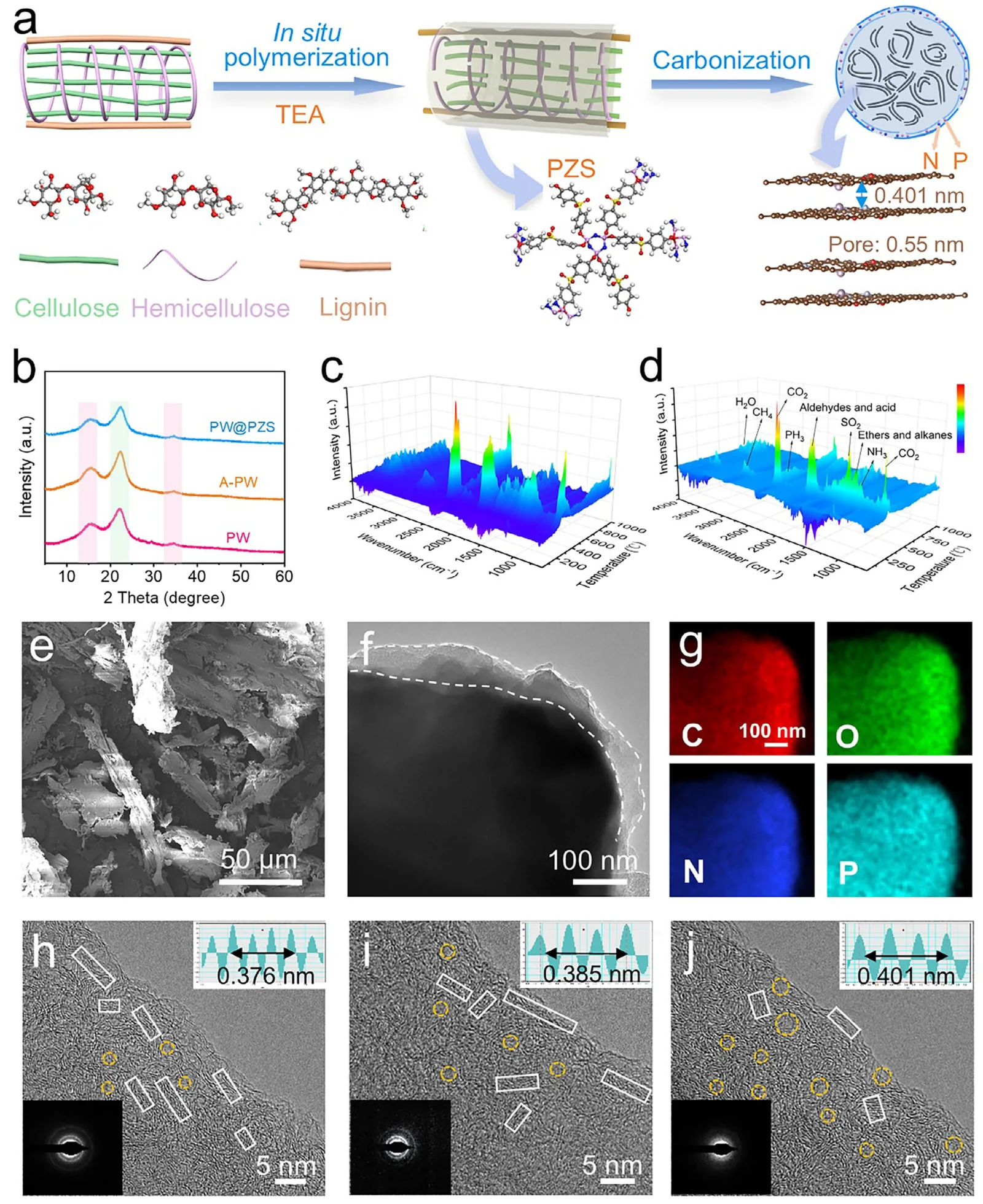
(**a**) Synthesis scheme of NP-HCs. (**b**) XRD patterns of the PW, A-PW, and PW@PZS precursors. In situ TGA-FTIR analysis results for (**c**) HCs and (**d**) NP-HCs. (**e**) SEM, (**f**) TEM images, and (**g**) elemental mappings of PW@PZS. HRTEM images of (**h**) HCs, (**i**) A-HCs, and (**j**) NP-HCs [76].

Fabrication strategies for heteroatom doping include in situ precursor co-doping, high-temperature post-doping via gas-phase treatment, liquid-phase oxidative modification, and plasma doping. Single-heteroatom doping suffers from evident limitations, whereas the synergy of two or more heteroatoms enables composite modulation of electronic structure, such as N-P co-doping. Meijuan Liu et al. prepared hard carbon anode materials (NP-HCs) with surface low-concentration N and P doping via an in situ conversion carbonization strategy, which exhibited significantly enhanced high-rate and LT sodium storage performance [76]. Figure 10 presents the synthesis process of NP-HCs along with the corresponding structural characterization results. Specifically, (a) is a schematic illustration of the in situ polymerization and carbonization synthesis of NP-HCs, clearly depicting the role of triethylamine, heteroatom doping, and the resulting interlayer spacing and pore structure characteristics; (b) shows the X-ray diffraction (XRD) patterns of poplar wood, acid-treated poplar wood, and polyphosphazene-coated poplar wood precursors, reflecting the influence of different pretreatments on precursor crystallinity; (c) and (d) present the in situ thermogravimetric analysis coupled with Fourier-transform infrared spectroscopy (TG-FTIR) results for hard carbon and NP-HCs, respectively, revealing their distinct pyrolysis pathways and gas evolution features; (e) and (f) are SEM and TEM images of polyphosphazene-coated poplar wood, and (g) displays the corresponding elemental mapping, confirming the homogeneous enrichment of nitrogen and phosphorus; (h), (i), and (j) are HRTEM images of hard carbon, acid-treated hard carbon, and NP-HCs, intuitively demonstrating that NP-HCs possess a larger interlayer spacing and a more abundant closed-pore structure.

More deeply, adjusting the coordination environment of transition metals to indirectly influence the occupancy state of sodium is also an effective way to optimize LT electrochemical performance. An optimized sodium environment helps improve ion diffusion rates and structural stability, providing a structural basis for reversible capacity retention under LT conditions.

### 5.3. Sodiophilic Interface Design and Pre-Sodiation Technology for Metal Anodes

Sodium metal anodes are regarded as one of the “ultimate” anode materials due to their highest theoretical specific capacity (1166 mAh g^−1^) and most negative electrode potential [77]. However, uncontrolled sodium dendrite growth and unstable SEI formation at low temperatures severely restrict their practical application.

Sodiophilic interface design introduces chemical components or three-dimensional structural hosts with high affinity for sodium to induce uniform sodium deposition and stripping. A review by Yunan Qin et al. systematically summarized several key sodiophilic design strategies and innovatively proposed carboxylate ester solvents as potential electrolyte components for LT SIBs [78].

Pre-sodiation technology is considered one of the most direct and effective methods to compensate for active sodium loss during the initial cycles of SIBs [79]. By pre-injecting additional Na^+^ into the positive/negative electrode materials via chemical/electrochemical means, the capacity loss caused by low ICE can be partially compensated, thereby enhancing the energy density and cycle life of full cells. The prevailing presodiation strategies are categorized into three routes: in situ anode presodiation, cathode presodiation, and electrolyte presodiation. Among them, the in situ anode presodiation method can be further divided into sacrificial salt pre-sodiation, electrochemical pre-sodiation, and chemical pre-sodiation [80]. However, pre-sodiation technology still faces technical bottlenecks in practical applications, such as uneven sodium distribution and difficulty in precisely controlling the degree of pre-sodiation, urgently requiring the development of more controllable and scalable novel pre-sodiation methods.

A sodiophilic interface alone merely optimizes sodium deposition morphology but cannot compensate for the initial irreversible sodium consumption. Direct presodiation on sodiophobic substrates inevitably induces severe sodium agglomeration and dendrite growth. The coupling of sodiophilic interface engineering and presodiation technology simultaneously suppresses sodium dendrite formation and particle agglomeration, as well as remedies the capacity loss of batteries.

## 6. Interface Regulation and Synergistic Optimization Strategies

### 6.1. SEI Composition Design and Construction of Inorganic-Rich Interfaces

The physicochemical properties of the SEI (SEI) directly determine the transport kinetics of sodium ions at the anode interface and the electrochemical stability of the electrode–electrolyte interface [8]. Under LT conditions, an ideal SEI should possess high ionic conductivity, low interfacial impedance, and good mechanical flexibility [81]. Theoretical calculations and experimental studies have shown that inorganic-rich SEI (rich in NaF, Na_2_CO_3_, Na_2_O, etc.) exhibits lower Na^+^ diffusion impedance and higher interfacial stability compared to organic-rich SEI [82].

Construction of inorganic-rich interfacial layers can be realized via three approaches: electrolyte solvation modulation, artificial prefabricated film fabrication, and intrinsic anode modification. These strategies directionally increase the proportion of inorganic crystalline phases within the SEI layer, constructing a passivation film with high ionic conductivity, superior structural stability and robust mechanical strength, which fundamentally addresses the sharp rise of interfacial impedance and rapid capacity degradation during cycling at LTs. Among these methods, electrolyte solvation modulation exhibits the highest practicability. By tuning the electrolyte composition, the solvent components undergo preferential decomposition during the initial charge–discharge cycle, in situ forming an SEI film abundant in target inorganic species. Yiwen Gao et al. confirmed that the electrode–electrolyte interface formed in the DMS-GN synergistic solvation system is rich in inorganic components such as NaF and Na_2_O, demonstrating higher stability and ionic conductivity [31]. This inorganic-rich interface effectively suppressed continuous electrolyte decomposition and active sodium loss, enabling NFM||HC full-cells to achieve improved cycling stability in the range of −40–45 °C. Anion-dominated solvation structures (i.e., close coordination between Na^+^ and anions) have been confirmed as a key precursor for forming inorganic-rich SEI. Therefore, regulating the degree of anion participation during desolvation through electrolyte formulation design has become a synergistic design paradigm that simultaneously optimizes ion transport kinetics and interfacial chemistry.

Artificial preformed films refer to a dense inorganic passivation layer fabricated directly on the anode surface via physical or chemical routes prior to electrolyte injection. Serving as the framework of artificial SEI, this layer thoroughly eliminates the drawbacks of inhomogeneous composition and loose structure of native SEI. In contrast, intrinsic anode modification tailors the surface chemical composition, defects and polarity of the anode to achieve active adsorption of film-forming precursors in the electrolyte. This strategy induces preferential nucleation and growth of inorganic phases on the electrode surface, thus generating an inorganic-rich SEI with enhanced interfacial adhesion strength.

### 6.2. Synergistic Regulation of Solvation Structure and Interfacial Chemistry

The intrinsic relationship between solvation structure and interfacial chemistry constitutes the core scientific issue in the design of LT SIBs [83,84]. In recent years, researchers have gradually realized that optimizing only the bulk properties of the electrolyte (e.g., ionic conductivity) or only the interfacial properties (e.g., SEI composition) is insufficient to achieve optimal LT performance; instead, the solvation structure and the interface formation process must be treated as an organically coupled whole for synergistic regulation [3,85].

This synergistic paradigm manifests at the molecular level: the solvation structure determines the energy dissipation of the desolvation process and the local concentration distribution of Na^+^ near the interface, while the chemical state of Na^+^ before and after reaching the electrode surface directly influences the nucleation and growth pathway of the SEI layer. Conversely, the chemical composition and structural features of the SEI in turn affect the subsequent desolvation and intercalation behavior of Na^+^. This bidirectional coupling implies that an excellent LT electrolyte design must balance reducing the desolvation energy barrier and inducing a stable SEI, rather than favoring one over the other [86].

The correlation between the solvation structure types of Na^+^ and interfacial chemistry is summarized in Table 3.

A review by M. Sai Bhargava Reddy et al. further expanded this framework, systematically analyzing the causes of LT performance degradation from the three dimensions of electrolyte, electrode, and interface, and emphasizing the critical significance of optimizing and integrating chemical design strategies across these three aspects for improving LT performance [2].

### 6.3. Synergistic Application of Advanced Characterization Methods and Theoretical Simulations

A deep understanding of the failure mechanisms and interfacial chemical evolution of SIBs at low temperatures relies on the synergistic support of advanced characterization techniques and theoretical simulations [9,87].

Spectroscopic methods (e.g., Raman spectroscopy, NMR) play an irreplaceable role in revealing LT solvation structures. Soohwan Kim et al. combining systematic molecular dynamics simulations, density functional theory calculations, experimental Raman spectroscopy, and NMR studies, deeply revealed the regulatory mechanism of different solvent ratios on the Na^+^ solvation structure and elucidated the influence of solvation structure on LT electrochemical performance [23]. X-ray photoelectron spectroscopy (XPS) and depth profiling provide key information on the chemical composition and vertical structure of the SEI, enabling researchers to understand SEI growth and evolution at the atomic level [88,89].

Furthermore, the rapid development of in situ/operando characterization techniques provides unprecedented capabilities to dynamically track interfacial evolution processes at low temperatures [90]. Techniques including cryogenic transmission electron microscopy (Cryo-TEM), in situ X-ray photoelectron spectroscopy (in situ XPS), time-of-flight secondary ion mass spectrometry (TOF-SIMS), and in situ electrochemical impedance spectroscopy can realize real-time observation of SEI nucleation, growth, thickness variation and compositional evolution, as well as quantitative analysis of interfacial failure behaviors during low-temperature operation and long-term cycling. Combining in situ LT characterization and machine learning-based data-driven strategies is expected to accelerate the intelligent design and screening of high-performance electrolytes and electrode materials, promoting the large-scale application of SIBs in harsh environments [91,92].

## 7. Conclusions and Outlook

SIBs, by virtue of their abundant sodium resources, cost advantages, and inherently favorable LT thermodynamic properties, are becoming a transformative technology for large-scale energy storage and LT electrochemical energy applications. Although significant progress has been made in recent years in the field of LT SIBs, several key scientific and technological bottlenecks remain to be overcome before practical application.

In terms of electrolytes, the development of low-freezing-point solvent systems has extended the lower operating temperature limit of SIBs to below −100 °C. However, balancing high-voltage stability and LT kinetics remains a core contradiction restricting the development of wide-temperature-range electrolytes. While weakly solvating strategies are effective in reducing the desolvation energy barrier, their sensitivity to temperature fluctuations requires future electrolyte designs to move toward more robust solvent-solvent interaction regulation pathways.

Regarding electrode materials, the three major cathode material families—layered oxides, polyanionic compounds, and PBAs—each exhibit their own advantages and limitations at low temperatures. Although modification strategies such as surface coating, ion doping, and microstructure engineering have achieved remarkable results, the scalability and long-term cycling stability of these strategies still require further validation. The design of hard carbon anodes is evolving from simple pore structure optimization to the synergistic regulation of electronic structure and interfacial chemistry, with the solvent co-intercalation strategy demonstrating a new approach to bypass the LT desolvation bottleneck.

In terms of interface regulation, the synergistic optimization of solvation structure and SEI chemistry has become the mainstream paradigm for LT SIB design. The construction of inorganic-rich SEI has shown unique advantages in improving LT interfacial kinetics, but the evolution and failure mechanisms of SEI during long-term cycling at low temperatures still require systematic study.

Looking forward, research on LT SIBs should continue to deepen in the following directions:

1. Development of ultra-low-temperature electrolyte systems. Although significant progress has been made, existing electrolyte systems still face the dual challenges of ionic conductivity decay and inadequate electrode compatibility in extremely cold environments below −60 °C. Novel systems based on high-entropy electrolytes and artificial anion receptors are expected to break through current limits.
2. Multi-scale material structural design. Establishing integrated optimization strategies spanning multiple scales—from atomic-scale doping site engineering to nanoscale pore regulation to micrometer-scale particle morphology design—is expected to significantly enhance LT kinetic performance.
3. Pre-sodiation technology and full-cell performance optimization. Pre-sodiation is a key pathway to compensate for first-cycle sodium loss and improve energy density. However, breakthroughs are still needed in terms of sodium distribution uniformity, cost control, and compatibility with existing battery manufacturing processes.
4. In situ characterization and machine learning-driven R&D paradigms. Combining in situ LT characterization to reveal solvation dynamics and interfacial evolution under realistic operating conditions, supplemented by machine learning-accelerated screening of electrolyte components and optimization of material structures, will significantly accelerate the R&D process of high-performance LT SIBs.

In summary, LT SIBs are at a critical transition period from fundamental research to engineering application. Bridging the gap from microscopic solvation structure design to macroscopic full-cell performance optimization and achieving systematic synergy among all components will be the core proposition for promoting the large-scale application of SIBs in extremely cold environments.

## Figures and Tables

**Table 1:** Comparison between LIBs and SIBs.

Category		LIBs	SIBs
Overview		High energy density; suitable for scenarios requiring high energy density, long cruising range and compact size.	High safety, excellent low-temperature performance and large capacity.
Cost		High cost. Lithium possesses low crustal abundance (~0.0065%) with uneven geographical distribution; copper foil (expensive) is mandatory as the anode current collector.	Low cost. Sodium features high crustal abundance (~2.75%) and global distribution; aluminium foil (low-cost) can be adopted for both anode and cathode current collectors.
Safety		Moderate to high risk; prone to thermal runaway.	Superior thermal stability and enhanced safety performance.
Technical indicators	Energy density	140–200 Wh/kg	100–175 Wh/kg
	Cycle life (times)	2000–6000+	2000–10,000+

**Table 2:** Single Heteroatom Doping.

Heteroatom	Doping Type	Evolution of Electronic Structure	Performance Improvement of Sodium Storage Anode	Drawbacks
N	N-type substitutional doping	Upshift of Fermi level, increased density of states, enhanced electrical conductivity	Improved rate capability, reduced LT impedance	Excessive doping increases specific surface area and decreases ICE
B	P-type substitutional doping	Generation of holes, narrowed band gap, accelerated interfacial charge transfer	Reduced interfacial impedance, more homogeneous and stable SEI film	High doping dosage damages the ordering of carbon framework and causes decay of plateau capacity
S	Large-atom substitutional doping	Distortion of carbon rings, enlarged interlayer spacing, strengthened electron delocalization	Low sodium intercalation resistance, stable structure during long-term cycling	Sulfur leaching easily occurs; trace parasitic gas evolution under high temperature and long cycling conditions
P	Interstitial & substitutional co-doping	Remarkable expansion of carbon interlayers, weakened interlayer interaction	Outstanding discharge performance under ultra-low temperature	High doping loading reduces the mechanical rigidity of carbon skeleton
O	Surface functional group doping	Enhanced surface polarity, localized dipole electric field	Greatly improved electrolyte wettability	Abundant oxygen-containing groups induce irreversible sodium consumption and sharp decline of ICE
F	Surface terminal passivation doping	Contraction of surface electron cloud, passivation of active edge sites	Suppressed side reactions, elevated ICE, inhibited gas generation	No sodium storage activity; excessive doping leads to decline in total specific capacity

**Table 3:** Na^+^ Solvation Structure.

Solvation Structure Type	Key Features	Interfacial Chemistry Consequences
SSIP (Solvent-Separated Ion Pairs; conventional low-concentration electrolyte)	Na^+^ fully encapsulated by solvent molecules; anions reside outside the first solvation shell	Solvent decomposition dominates SEI formation; high proportion of organic components; poor structural stability; sluggish desolvation kinetics
CIP (Contact Ion Pairs; elevated concentration or weakly solvating system)	One or more anions enter the first solvation shell and directly coordinate with Na^+^	Anions begin to participate in film formation; increased content of inorganic components in SEI
AGG (Ionic Aggregates; high concentration/locally high concentration)	Na^+^ and anions form network-like aggregates; solvent molecules are excluded from the solvation shell	Anions undergo preferential decomposition; ultrathin, dense, and inorganic-rich SEI/CEI is formed; extremely low desolvation energy

## Data Availability

No new data were created or analyzed in this study. Data sharing is not applicable to this article.
